# Nasal airflow of patient with septal deviation and allergy rhinitis

**DOI:** 10.1186/s42492-021-00080-2

**Published:** 2021-05-20

**Authors:** Zi Fen Lim, Parvathy Rajendran, Muhamad Yusri Musa, Chih Fang Lee

**Affiliations:** 1 School of Aerospace Engineering, Universiti Sains Malaysia, 11800 Pulau Pinang, Malaysia; 2 Faculty of Engineering & Computing, First City University College, 47800 Selangor, Malaysia; 3 Advanced Medical and Dental Institute, Universiti Sains Malaysia, 11800 Pulau Pinang, Malaysia

**Keywords:** Three-dimensional nasal airflow model, Septal deviation, Allergy rhinitis, Computational fluid dynamics

## Abstract

A numerical simulation of a patient’s nasal airflow was developed via computational fluid dynamics. Accordingly, computerized tomography scans of a patient with septal deviation and allergic rhinitis were obtained. The three-dimensional (3D) nasal model was designed using InVesalius 3.0, which was then imported to (computer aided 3D interactive application) CATIA V5 for modification, and finally to analysis system (ANSYS) flow oriented logistics upgrade for enterprise networks (FLUENT) to obtain the numerical solution. The velocity contours of the cross-sectional area were analyzed on four main surfaces: the vestibule, nasal valve, middle turbinate, and nasopharynx. The pressure and velocity characteristics were assessed at both laminar and turbulent mass flow rates for both the standardized and the patient’s model nasal cavity. The developed model of the patient is approximately half the size of the standardized model; hence, its velocity was approximately two times more than that of the standardized model.

## Introduction

The nasal cavity is one of the most critical parts of the human respiratory system [1–3]. Nasal obstructions, such as nasal septum deviations, enlarged turbinates, nasal polyps, enlarged adenoids, tumors, and nasal congestion, can trigger breathing difficulties. In this study, two major nasal obstructions are considered: septal deviation and allergic rhinitis.

The nasal septum is the bone that divides one side of the nose from the other. It is rarely perfectly straight, and it is comprises a central supporting skeleton covered on each side by mucous membranes [4, 5]. The front part of this natural partition is a firm but bendable structure, made mostly of cartilage and covered by skin with a substantial supply of blood vessels. In addition, it is slightly crooked in over 80% of people [6].

When the septum is crooked or deviated, it blocks nasal passage, and a surgical operation, submucosal resection, is required to restore clear breathing. Septal deviations play a critical role in nasal obstruction symptoms, the aesthetic appearance of the nose, increased nasal resistance, and sometimes snoring [7]. Symptoms of a deviated septum include sinus infections, sleep apnea, snoring, repetitive sneezing, facial pain, nosebleeds, and difficulty with breathing, as well as mild to severe loss of smell [8].

Rhinitis is defined as an inflammation of the nasal mucosa, which affects approximately 40% of the population [9–11]. Allergic rhinitis is the most common cause of mucosal inflammation, and it affects one in six individuals [12]. There are two types of allergic rhinitis: seasonal and perennial. Seasonal allergic rhinitis can occur in spring, summer, and early fall. It is usually caused by allergic sensitivity to airborne mold spores or pollen from grass, trees, and weeds. Allergy rhinitis is estimated to affect nearly one in every six Americans, and generates $2 to $5 billion in direct health expenditures annually [12].

To better understand the physiology of the nasal cavity, this study adopts the computational fluid dynamics (CFD) method to obtain and compare flow patterns. Hence, CFD has become a fast and convenient research tool for studying airflow in the human airway, especially when investigating heat and humidity transfer, which is difficult to investigate with other experimental techniques [13–16]. CFD simulations help to better understand the complex anatomy of the nasal, as well as the implications of disease and surgery. It has the potential to help surgeons and rhinologists plan surgery and simulate surgery by correcting perceived anatomical abnormalities on a model called “virtual surgery” and then comparing flow predictions to help surgeons and rhinologists decide whether minor or major corrective surgery is needed [17]. Hence, CFD simulation findings, such as cross-sectional areas, velocity magnitudes, contours, and streamlines, can be examined in detail, allowing for improvements and corrections if there are any inadequacies or insufficiencies in information for rhinologists as a pre-operative tool to aid in clinical decision making.

In this study, the computerized tomography (CT) scans of a female adult patient with septal deviation and allergic rhinitis were obtained with consent from the Advanced Medical and Dental Institute, University of Science Malaysia. The three-dimensional (3D) model of the nasal cavity was developed from CT scans and exported to CATIA V5, and then airflow simulation was performed. To analyze the impact of septal deviation and allergic rhinitis on nasal airflow, the obtained results will be compared with a standardized nasal cavity.

## Methods

CFD can predict airflow and particle deposition in the nasal cavity [11, 18–21]; hence, it is widely used in the airflow prediction of complex structures. In this case, FLUENT was adopted for the simulation to obtain an accurate airflow simulation of the patient’s nasal cavity. To determine the impact of septal deviation and allergic rhinitis, the CFD results of fluid mechanical properties were then analyzed and compared with the standardized nasal cavity model of a health female adult.

### Model reconstruction

InVesalius 3.0 is an open-source software for virtual modeling, and it can obtain an accurate model of the anatomical region to be studied, as high-quality medical images are necessary [22]. The digital imaging and communications in medicine files of a female patient were imported into the InVesalius 3.0. The number of two-dimensional (2D) slices in three axes is presented in Table 1. Because this study focused solely on the nasal cavity, the number of slices that did not involve the nasal cavity was not considered.

**Table 1:** Number of 2D slices for CT scans of patient

Axis	Total number of slices	Number of slices used
Axial	238	103–171
Coronal	511	216–427
Sagittal	511	215–301

An inverted model of the patient’s airway was constructed by filling the space in which the air flows, including the 2D slices mentioned in Table 1. The reconstructed airway of the subject can be visualized using this software. Then, the model was exported as a STL file and imported into CATIA V5 as the cloud point. The model was then smoothened and modified after mesh creation in CATIA. Surface and volume creation will also be performed in CATIA to reconstruct a solid model. Then, the 3D model saved as a STEP file was imported into ANSYS FLUENT for simulation.

### Simulation

**Fig. 1:**
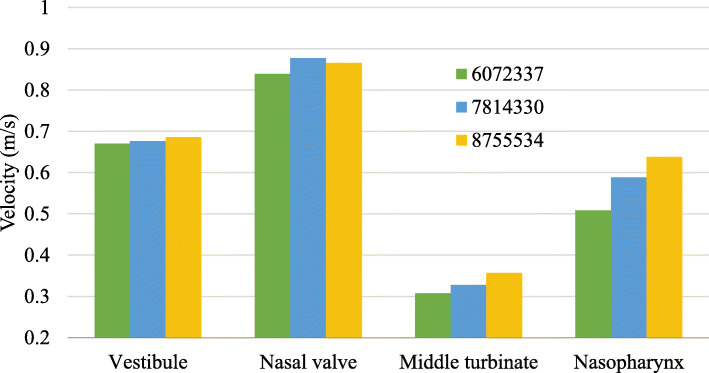
Mesh dependency study at mass flow rate of 125 mL/s

The nasal wall was assumed to be rigid, with a no-slip boundary condition, and mucous effects were assumed to be negligible [23, 24]. The nostril inlet is defined by the mass flow inlet, while the outflow boundary condition represents the outlet at the nasopharynx. Any backflow at the outlet was assumed to be at 32.6 °C, and 100% relative humidity was imported into ANSYS FLUENT [15]. The pressure-based model was adopted for this simulation, as the density of air was assumed to be constant throughout the geometry [25]. The flow of mucus was not considered owing to its minimal thickness and low velocity [23, 26]. The mesh models of the three meshing types for the mesh dependency study (Fig. 1) and its model elements are presented in Table 2.

**Table 2:** Meshing of model

Meshed model	Meshing type	Number of elements
	Coarse	6,072,337
	Medium	7,814,330
	Fine	8,755,534

The accuracy of the numerical results is closely related to the mesh density, as well as its distribution. Therefore, the mesh plays a significant role in the outcome of numerical simulations [10, 27]. A functional mesh must be able to resolve the velocity vectors and effectively capture the fluid properties in all regions inside the nasal cavity [28, 29]. After the mesh dependency study, a simulation was performed for the medium meshed model in ANSYS FLUENT. The mesh dependency study exhibited an optimized meshing of 7,814,330 elements. The model was adopted for mass flow rates ranging from 100 mL/s to 425 mL/s, as the inspiratory flow rate for healthy adults is varied between 80 mL/s and 200 mL/s for light breathing and a range of 200–660 mL/s for non-normal conditions such as during exercises [30].

## Results

The results obtained from the simulation are presented and discussed in three sections. The Geometrical comparisons section presents a geometrical comparison of two nasal cavity models: the current study with the female adult-patient model and the standardized female adult model generated by Lee et al. [31] in their previous research. In this section, the comparison is performed via the visual observation of both the 3D nasal cavity models and their cross-sectional areas at different planes. The Pressure section focuses on the pressure at different planes with varying mass flow rates, and the decrease in pressure of the nasal model. The Velocity section then compares both models, including the velocity magnitude in different planes and graphical results obtained from the CFD analysis, as well as the velocity contour of both models.

### Geometrical comparisons

Respiratory physiology and pathology significantly depend on the airflow inside the nasal cavity. Because nasal airflow is profoundly affected by the geometry of the flow passage, changes in the shape of the nasal cavity due to diseases or surgical treatments alter the nasal resistance and functions of the nose [32–35]. Geometric configuration plays a significant role in the flow distribution inside the nasal cavity, especially in disease cases, as the imbalance of the nasal cavity owing to septal deviation is considered to be a common etiology of nasal airway obstruction [4, 13, 16]. The geometric comparisons of the 3D models are presented in Table 3.

**Table 3:** Geometrical comparisons of 3D models

Nasal model	3D geometry
Female adult patient with septal deviation and allergic rhinitis	
Standardized female adult by Lee et al. [31]	

As presented in Table 3, the model of the patient’s nasal cavity exhibits several disconnections that cause difficulties in breathing [15, 36]. In addition, the cross-section of the middle turbinate significantly differs from the standardized model in size, and the standardized model has a smoother and cleaner airway than that of the patient’s model. The patient’s model shows many creases on the surface as there are several growths in the nasal airway owing to allergic rhinitis [9, 37, 38].

For better observations, the comparisons are focused on four different cross-sectional areas: vestibule, nasal valve, middle turbinate, and nasopharynx. To demonstrate the differences due to septal deviation and allergic rhinitis, the cross-sectional areas at different planes of both models were compared and are presented in Table 4.

**Table 4:** Comparison between cross-sectional areas at different planes of both models

Plane	Cross-sectional area (cm^2^)		Percentage difference
	Standardized model by Lee et al. [31]	Patient’s model	
Inlet	0.34872949	0.15066816	57%
Vestibule	0.35425289	0.15388511	57%
Nasal valve	0.29106226	0.13621432	53%
Middle turbinate	0.64515747	0.26335791	59%
Nasopharynx	0.54266273	0.19838503	63%
Outlet	0.35474969	0.16834382	53%
Average	0.422769088	0.178475725	58%

**Fig. 2:**
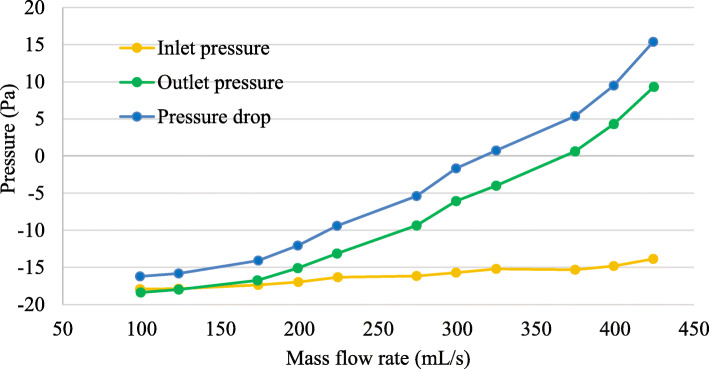
Graph of pressure drop against mass flow rate

In general, the standardized model by Lee et al. [31] exhibits a higher cross-sectional area than the patient’s model. On average, the patient’s model is 58% smaller than the standardized model. The percentage differences in each plane ranges from 53% to 63%, which indicates that the patient’s model is smaller in size than that of the standardized model. As presented in Fig. 2, both models exhibit a similar trend in their cross-sectional areas. In addition, the smallest and largest cross-sectional areas of both models are at their nasal valve and middle turbinate, respectively.

To obtain a more accurate figure that demonstrates the apparent differences between both models, the volume of each model was also calculated. The volume of the patient’s model is 23.71 cm^3^, whereas that of the standardized model is 45.23 cm^3^. Therefore, the volume of the patient’s model is 48% smaller than that of the standardized model. The cross-sectional area and volume results are relatively similar to the patient’s model, which is approximately half the size of the standardized model.

### Pressure

To prevent diseases and determine their treatment methods, it is necessary to understand the breathing mechanism [39, 40]. In several studies, the pressure of airways is conventionally measured as a function of time at the domain exit [40, 41]. Therefore, a computational analysis was performed on the patient’s 3D nasal model, and the pressure drop was calculated for mass flow rates ranging from 100 to 425 mL/s. The pressure obtained for this part is the pressure relative to the atmospheric pressure. Here, negative pressure indicates human breathing with a pressure lower than atmospheric pressure. The pressure drop across the nasal cavity, from the model inlet to its outlet, was obtained, as illustrated in Fig. 2.

**Fig. 3:**
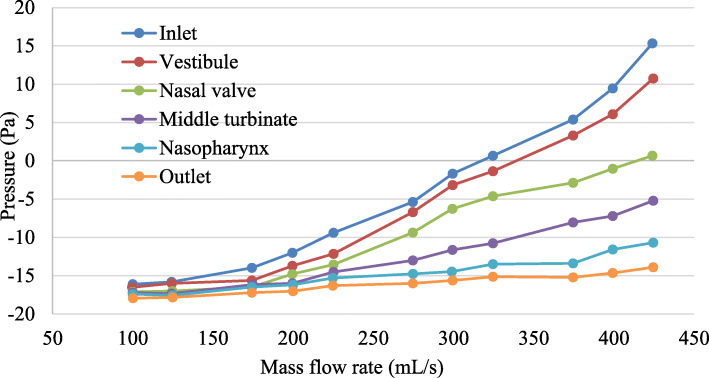
Pressure on different planes at different mass flow rate

The inlet pressure increased drastically as the mass flow rate increased, whereas the outlet pressure remained constant at different mass flow rates. From the obtained graph, it can be observed that the pressure drop increases gradually as the mass flow rate increases. The pressure difference induces different local flow rates and wall shear stress distributions, thus triggering further local dynamics [41, 42]. To observe the pressure changes throughout the nasal airway, the pressures at different planes and different mass flow rates are obtained, as presented in Fig. 3.

The graph shows that the pressure drop increases steadily throughout the nasal airway owing to the wall shear stress [41, 42]. The wall shear stress during inspiration was predominantly higher in the anterior region than in other regions [43]. After a mass flow rate of 250 mL/s, the pressure change increases drastically, and some researchers have stated that a mass flow rate of less than 250 mL/s is defined as laminar airflow. In comparison, a mass flow rate higher than 250 mL/s is considered turbulent airflow [25, 43, 44]. The resistance in the airways triggers a pressure drop. Airway resistance is due to the flow triggered by frictional forces. It is defined as the ratio of the driving pressure to the airflow rate.

Resistance to airflow in the airways depends on (1) the flow (laminar or turbulent flow), (2) dimensions of the airway, and (3) viscosity of the gas [39]. Therefore, pressure drops climactically at the turbulent flow. It is also apparent that the pressure continues to drop from the inlet to the outlet at the same flow rate. Inlet pressure is always the highest pressure, followed by pressures at the vestibule, nasal valve, middle turbinate, nasopharynx, and finally, the nasal outlet, which has the lowest pressure. The pressure contour obtained from the side view in Table 5 depicts the pressure distribution for both laminar and turbulent flows.

**Table 5:** Side view pressure contour for both laminar and turbulent flows

Mass flow rate	125 mL/s	400 mL/s
Type of flow	Laminar	Turbulent
		

### Velocity

The magnitude of velocity between the patient’s model and the standardized model by Lee et al. [31] were compared at different planes. The velocity magnitude of the models at the same mass flow rate, 125 mL/s, is presented with percentage differences in Table 6, and plotted as a graph to demonstrate the velocity characteristics of the patient model. The results obtained are also presented in Table 6.

**Table 6:** Comparison between velocity magnitude at different planes at 125 mL/s

Planes	Velocity magnitude (m/s)		Percentage different
	Standardized model by Lee et al. [31]	Patient’s model	
Inlet	0.38773257	0.796214	−105%
Vestibule	0.35802209	0.675221	−89%
Nasal valve	0.42031079	0.880186	−109%
Middle turbinate	0.19572208	0.331966	−70%
Nasopharynx	0.25819995	0.589943	−128%
Outlet	0.35236112	0.642273	−82%
Average	0.328724767	0.652633833	−99%

In general, the velocity magnitude of the patient’s model is significantly higher than that of the standardized model, with percentage differences ranging from 70% to 128%, and an average of 99% higher velocity than the standardized model. The lowest differences in velocity for both models are at the middle turbinate. Therefore, septal deviation and allergic rhinitis had the least effect on the middle turbinate.

However, the highest velocity difference occurs at the nasopharynx, which cleans the inspired air of pollutant particles and protects the delicate lower respiratory tract. Therefore, a high velocity at this plane does not cause any inconvenience to the patient [45]. From the graph, it is evident that both models have the same velocity characteristics throughout the inhalation process. Both models exhibit their highest and lowest velocities at the nasal valve and middle turbinate, respectively.

The primary function of the nasal valve is to limit the amount of airflow generated in the nasal cavity and to converge the flow. Therefore, the nasal valves always require a high velocity before the separate airflows converge from two to one. Because the middle turbinate has the largest surface area, it decreases the erectile and vascular tissue density, and is less prominent in nasal airflow patterns. Therefore, the middle turbinates always have the lowest air velocity magnitude throughout the nasal cavity [46].

To observe the differences in velocity distribution for both laminar and turbulent flows, the velocity contour and velocity vector of the side view for both airflow types are presented in Table 7. The model for the turbulent flow distribution exhibits a higher radial flow after the middle turbinate than the model for the laminar flow distribution. This verifies that there are significant differences in the velocity pattern characteristics for both laminar and turbulent flows.

**Table 7:** Side view of velocity contour and vector for both laminar and turbulent flows

Mass flow rate	Type of flow	Side view of velocity contour	
125 mL/s	Laminar		
400 mL/s	Turbulent		

The flow distributions for both laminar and turbulent flows are almost the same at the beginning. However, differences emerge after the middle turbinate, as it has the most complex structure throughout the nasal cavity. Vortices occur after the middle turbinate because the middle and inferior turbinates are crucial structures for filtration and are used to enhance heating and humidification, when the mucosal wall surface area is enlarged [47].

The velocity vectors and contours are presented in Table 7 to compare the differences between the laminar and turbulent flows of 125 mL/s and 400 mL/s, respectively. Both models exhibit similar patterns of velocity contours, with significant differences in velocity magnitudes. The higher mass flow rate of the turbulent flow increases the magnitudes of velocity observed along the nasal cavity. However, the nasal valve usually has the highest velocity magnitude for both laminar and turbulent flows. Simultaneously, this validates the role of the nasal valve in converging airflow from two to one, as well as that of the middle turbinate in heating and humidifying the air owing to the increase in the surface area of the meatus regions [47].

The velocity contour was considered for comparison. It clearly illustrates the physical differences between both models, as it presented 2D velocity fields in planes parallel to the flow pattern throughout the nasal cavity [41, 48]. The airflow through each of these regions was computed by integrating the axial component of the velocity with the coronal cross-section. These flow volumes were used to estimate the relative flow allocation within each cross-section region as a percentage of the total volumetric flow for that cross-section [49]. Considering the steady airflow, sequentially recorded data could be used to determine the location of errors with magnitudes relative to their respective velocity vectors [41, 50]. It is necessary to ensure a no-slip condition at all solid boundaries, which means that the contour plots must be zero at all physical limitations [41, 48]. A comparison between the velocity contours at a mass flow rate of 125 mL/s is presented in Table 8.

**Table 8:** Comparison of velocity contours at mass flow rate 125 mL/s

Planes	Standardized model by Lee et al. [31]		Patient’s model	
Vestibule				
Nasal valve				
Middle turbinate				
Nasopharynx				

The velocity distribution inside the airway can be observed from the obtained velocity contours [31, 51]. The velocity of the airflow and its spatial and temporal variations close to the wall, as well as corresponding shear rate or shear stress at the wall are essential factors necessary for several physiological processes, such as the pressure drop throughout the nose, particle deposition, and exchange processes at the wall [16, 51, 52]. Wall shear has also been identified as a putative agent for the mechano-transduction between the airflow and nasal epithelium [52, 53].

The standardized model has an excellent oval shape for both the vestibule and nasal valve; however, the patient’s model has an irregular and inconsistent shape. The shape irregularity at the septal deviation caused the air to enter unevenly. The middle turbinate of the standardized model is almost symmetrical on the left and right sides, and it has a broader airway. However, the middle turbinate of the patient model is narrower and unsymmetrical compared to the standardized model. This irregularity is due to the airway’s growth towing to allergic rhinitis, which reduces the patient’s nasal airway [9, 15]. The velocity of the standardized model ranged from 0 to 0.852 m/s, whereas that of the patient’s model ranged from 0 to 1.84 m/s. The standardized model has a smaller range in velocity than the patient’s model, as it has a broader airway, while the patient’s model has overall higher velocity magnitudes than the standardized model, during breathing.

## Discussion

A summary of clinical implications and translations for this nasal airflow study is presented in Table 9. Therefore, using the 3D reconstruction model with airflow analysis as presented in this paper, specialists can see a clear picture of patients’ bodies, enabling a better understanding of the condition, which helps foresee a patient’s body responds to illness treatment. Moreover, these 3D reconstruction models are useful in the rapid prototyping technology of modified anatomical implants and demonstrate multiple abnormalities requiring an additional diagnostic value. The outcome of the study may also minimize disease risk through preventive medicine and conventional drug therapies.

**Table 9:** A summary of clinical implications

Division	Standardized model by Lee et al. [31]	Patient’s model
Nasal airway surface	A smooth and clean airway	Lots of creases on the surface as there are many growths in the nasal airway due to allergy rhinitis; Many disconnections that lead to difficulties in breathing.
Cross-sectional areas	Good nasal cross-sectional areas	Averagely 58% smaller cross-sectional areas than the standardized model.
	Smallest cross-sectional area at the nasal valve and the largest cross-sectional area in the middle turbinate.	
Volume	Good nasal volume	Forty-eight percent less volume than the standardized model
Nasal pressure	Pressure drop increases gradually throughout the nasal airway due to the wall shear stress; Wall shear stress during inspiration is predominantly higher in the anterior region; Inlet pressure has always been the highest, followed by the vestibule, nasal valve, middle turbinate, nasopharynx, and finally, the nasal outlet with the lowest pressure.	
Velocity	Good nasal velocity magnitude	The patient’s model’s overall velocity magnitude is much higher than the standardized model, ranged from 70% to 128% of differences and have an average of 99% higher velocity than the standardized model; Septal deviation and allergy rhinitis cause the least effect on the middle turbinate; High velocity at the nasopharynx does not cause any inconvenience for the patient.
	The lowest differences in velocity are at the middle turbinate; The highest velocity difference occurs at the nasopharynx to clean the inspired air of pollutant particles and protect the delicate lower respiratory tract.	
Velocity contour	The flow distributions for both laminar and turbulent are almost the same at the beginning; Turbulent flow distribution has more radial flow after the middle turbinate.	
Shape	Excellent oval shape for both the vestibule and nasal valve; Middle turbinate is almost symmetry for the left and right side.	Has irregular and inconsistent shapes; Shape irregularity at the septal deviation that caused the air to enter unevenly; Middle turbinate is narrower and unsymmetrical.

## Conclusions

In conclusion, a 3D nasal cavity model of a female adult patient with septal deviation and allergic rhinitis was developed. The airflow characteristics owing to septal deviation and allergic rhinitis were investigated and studied by analyzing the airflow simulation results using ANSYS Fluent. The developed model for the patient is approximately half the size of the standardized model; hence, its velocity is approximately two times higher than that of the standardized model during inspiration. Comparisons were carried out to study the impact of septal deviation and allergic rhinitis on the patient’s nasal airflow. The standardized model with a smoother and wider airway exhibited a better velocity distribution during breathing than the patient’s model. In contrast, the patient’s model with growth blockage had an asymmetrical and narrow airway, thereby causing the patient’s breathing to be higher in velocity than that of the healthy standardized model, at the same mass flow rate. Using the presented model, specialists can understand the human form in three dimensions, which allows them to predict how the body will respond to illness better. Furthermore, these 3D reconstruction models can be used in the rapid prototyping of modified anatomical implants and can show multiple abnormalities, which adds to the diagnostic value. The study’s findings may also help to reduce disease risk through preventive medicine and traditional drug therapies.

## Data Availability

All data generated or analyzed during this study are included in this published article.

## Abbreviations

2D: Two-dimensional
3D: Three-dimensional
ANSYS: Analysis system
CATIA: Computer aided three-dimensional interactive application
CFD: Computational fluid dynamics
CT: Computerized tomography
FLUENT: Flow oriented Logistics Upgrade for Enterprise Networks

## References

[1] Hansen F, Wood DE (2013). The adrenal fatigue solution.

[2] Marks TN, Maddux SD, Butaric LN, Franciscus RG (2019). Climatic adaptation in human inferior nasal turbinate morphology: evidence from Arctic and equatorial populations. Am J Phys Anthropol.

[3] Tracy LF, Basu S, Shah PV, Frank-Ito DO, Das S, Zanation AM (2019). Impact of endoscopic craniofacial resection on simulated nasal airflow and heat transport. Int Forum Allergy Rhinol.

[4] Kim SK, Heo GE, Seo A, Na Y, Chung SK (2014). Correlation between nasal airflow characteristics and clinical relevance of nasal septal deviation to nasal airway obstruction. Respir Physiol Neurobiol.

[5] Radulesco T, Meister L, Bouchet G, Varoquaux A, Giordano J, Mancini J (2019). Correlations between computational fluid dynamics and clinical evaluation of nasal airway obstruction due to septal deviation: an observational study. Clin Otolaryngol.

[6] Deviated Septum (2021). American academy of otolaryngology-head and neck surgery.

[7] Teixeira J, Certal V, Chang ET, Camacho M (2016). Nasal septal deviations: a systematic review of classification systems. Plast Surg Int.

[8] Holbrook EH, Meyers AD (2021). Disorders of taste and smell.

[9] Solé D, Sakano E, Cruz AA, Pastorino A, Prado E, de Mello FCF (2012). III Consenso Brasileiro sobre Rinites. Braz J Otorhinolaryngol.

[10] Borojeni AAT, Garcia GJM, Moghaddam MG, Frank-Ito DO, Kimbell JS, Laud PW (2020). Normative ranges of nasal airflow variables in healthy adults. Int J Comput Assist Radiol Surg.

[11] Zhang Y, Zhou XD, Lou M, Gong MJ, Zhang JB, Ma RP (2019). Computational fluid dynamics (CFD) investigation of aerodynamic characters inside nasal cavity towards surgical treatments for secondary atrophic rhinitis. Math Probl Eng.

[12] Schoenfeld BJ, Contreras B, Tiryaki-Sonmez G, Wilson JM, Kolber MJ, Peterson MD (2015). Regional differences in muscle activation during hamstrings exercise. J Strength Cond Res.

[13] Kim SK, Na Y, Kim JI, Chung SK (2013). Patient specific CFD models of nasal airflow: overview of methods and challenges. J Biomech.

[14] Bruening J, Goubergrits L, Hildebrandt T (2016). Team 190: CFD simulation of airflow within a nasal cavity The UberCloud.

[15] Garcia GJM, Bailie N, Martins DA, Kimbell JS (2007). Atrophic rhinitis: a CFD study of air conditioning in the nasal cavity. J Appl Physiol.

[16] Faramarzi M, Baradaranfar MH, Abouali O, Atighechi S, Ahmadi G, Farhadi P (2014). Numerical investigation of the flow field in realistic nasal septal perforation geometry. Allergy Rhinol (Providence).

[17] Bailie N, Hanna B, Watterson J, Gallagher G (2006). An overview of numerical modelling of nasal airflow. Rhinology.

[18] Lin CL, Tawhai MH, McLennan G, Hoffman EA (2009). Multiscale simulation of gas flow in subject-specific models of the human lung. IEEE Eng Med Biol Mag.

[19] Nomura T, Ushio M, Kondo K, Kikuchi S (2018). Effects of nasal septum perforation repair on nasal airflow: an analysis using computational fluid dynamics on preoperative and postoperative three-dimensional models. Auris Nasus Larynx.

[20] Borojeni AAT, Frank-Ito DO, Kimbell JS, Rhee JS, Garcia GJM (2017). Creation of an idealized nasopharynx geometry for accurate computational fluid dynamics simulations of nasal airflow in patient-specific models lacking the nasopharynx anatomy. Int J Numer Method Biomed Eng.

[21] Li LF, Zang HR, Han DM, Ramanathan M, Carrau RL, London NR (2020). Impact of a concha bullosa on nasal airflow characteristics in the setting of nasal septal deviation: a computational fluid dynamics analysis. Am J Rhinol Allergy.

[22] Camilo AA, Amorim PHJ, Moraes TF, de S. Azevedo F, da Silva JVL (2012) Invesalius: medical image edition. Paper presented at the 1st international conference on design and processes for medical devices, West Garda Hotel, Brescia, 2-4 May 2012

[23] Ruiz CP, Ruiz CF, López AC, Español CC (2005). Computational fluid dynamics simulations of the airflow in the human nasal cavity. Acta Otorrinolaringol Esp.

[24] Wang DY, Lee HP, Gordon BR (2012). Impacts of fluid dynamics simulation in study of nasal airflow physiology and pathophysiology in realistic human three-dimensional nose models. Clin Exp Otorhinolaryngol.

[25] Smith K (2008). CFD analysis of pressure and flow characteristics of the human nose.

[26] Farzal Z, Del Signore AG, Zanation AM, Ebert JC, Frank-Ito D, Kimbell JS (2019). A computational fluid dynamics analysis of the effects of size and shape of anterior nasal septal perforations. Rhinology.

[27] Zubair M, Abdullah MZ, Ahmad KA (2013). Hybrid mesh for nasal airflow studies. Comput Math Methods Med.

[28] Cheng YS, Yeh HC, Guilmette RA, Simpson SQ, Cheng KH, Swift DL (1996). Nasal deposition of ultrafine particles in human volunteers and its relationship to airway geometry. Aerosol Sci Technol.

[29] Mylavarapu G, Murugappan S, Mihaescu M, Kalra M, Khosla S, Gutmark E (2009). Validation of computational fluid dynamics methodology used for human upper airway flow simulations. J Biomech.

[30] Wen J, Inthavong K, Tu JY, Wang SM (2008). Numerical simulations for detailed airflow dynamics in a human nasal cavity. Respir Physiol Neurobiol.

[31] Lee CF, Abdullah MZ, Ahmad KA, Shuaib IL (2013). Standardization of malaysian adult female nasal cavity. Comput Math Methods Med.

[32] Lee KB, Jeon YS, Chung SK, Kim SK (2016). Effects of partial middle turbinectomy with varying resection volume and location on nasal functions and airflow characteristics by CFD. Comput Biol Med.

[33] Frank-Ito DO, Kimbell JS, Borojeni AAT, Garcia GJM, Rhee JS (2019). A hierarchical stepwise approach to evaluate nasal patency after virtual surgery for nasal airway obstruction. Clin Biomech.

[34] Li C, Sun XZ, Zhao M, Yu S, Huang Q, Zhang XQ (2019). Comparison of airflow characteristics after Draf III frontal sinus surgery and normal person by numerical simulation. Math Biosci Eng.

[35] Hildebrandt T, Brüning JJ, Schmidt NL, Lamecker H, Heppt W, Zachow S (2019). The healthy nasal cavity-characteristics of morphology and related airflow based on a statistical shape model viewed from a surgeon’s perspective. Facial Plast Surg.

[36] Dilek F, Ozkaya E, Gultepe B, Yazici M, Iraz M (2016). Nasal fluid secretory immunoglobulin a levels in children with allergic rhinitis. Int J Pediatr Otorhinolaryngol.

[37] Hamerschmidt R, Hamerschmidt R, Moreira ATR, Tenorio SB, Timi JRR (2016). Comparison of turbinoplasty surgery efficacy in patients with and without allergic rhinitis. Braz J Otorhinolaryngol.

[38] Kutlug S, Gunbey E, Sogut A, Celiksoy MH, Kardas S, Yildirim U (2016). Evaluation of olfactory function in children with allergic rhinitis and nonallergic rhinitis. Int J Pediatr Otorhinolaryngol.

[39] Chovancová M, Elcner J (2014). The pressure gradient in the human respiratory tract. EPJ Web Conf.

[40] Kim JH, Roberge RJ, Powell JB, Shaffer RE, Ylitalo CM, Sebastian JM (2015). Pressure drop of filtering facepiece respirators: how low should we go?. Int J Occup Med Environ Health.

[41] Lee JH, Na Y, Kim SK, Chung SK (2010). Unsteady flow characteristics through a human nasal airway. Respir Physiol Neurobiol.

[42] Rapoport D, Norman R, Nielson M (2001). Nasal pressure airflow measurement.

[43] Riazuddin VN, Zubair M, Abdullah MZ, Ismail R, Shuaib IL, Hamid SA (2011). Numerical study of inspiratory and expiratory flow in a human nasal cavity. J Med Biol Eng.

[44] Lee CF, Abdullah MZ, Ahmad KA, Shuaib IL (2014). Analytical comparisons of standardized nasal cavity. J Med Imaging Health Inf.

[45] Li HF, Tian ZF, Tu JY, Yang W, Yeoh GH, Xue CL (2006). Studies of airflow through a human nasopharynx and pharynx airway. Abstracts of the 5th international conference on CFD in the process industries, CSIRO, Melbourne, 13-15 December 2006.

[46] Cook PR, Begegni A, Bryant WC, Davis WE (1995). Effect of partial middle turbinectomy on nasal airflow and resistance. Otolaryngol Head Neck Surg.

[47] Wen J, Inthavong K, Tian ZF, Tu JY, Xue CL, Li CG (2007). Airflow patterns in both sides of a realistic human nasal cavity for laminar and turbulent condition. Abstracts of the 16th Australasian fluid mechanics conference, School of Engineering, the University of Queensland, Brisbane, 3-7 December 2007.

[48] Garcia GJM, Hariri BM, Patel RG, Rhee JS (2016). The relationship between nasal resistance to airflow and the airspace minimal cross-sectional area. J Biomech.

[49] Segal RA, Kepler GM, Kimbell JS (2008). Effects of differences in nasal anatomy on airflow distribution: a comparison of four individuals at rest. Ann Biomed Eng.

[50] Kelly JT, Prasad AK, Wexler AS (2000). Detailed flow patterns in the nasal cavity. J Appl Physiol.

[51] Doorly DJ, Taylor DJ, Schroter RC (2008). Mechanics of airflow in the human nasal airways. Respir Physiol Neurobiol.

[52] Elad D, Wolf M, Keck T (2008). Air-conditioning in the human nasal cavity. Respir Physiol Neurobiol.

[53] Elad D, Naftali S, Rosenfeld M, Wolf M (2006). Physical stresses at the air-wall interface of the human nasal cavity during breathing. J Appl Physiol.

